# Gene silencing of TACE enhances plaque stability and improves vascular remodeling in a rabbit model of atherosclerosis

**DOI:** 10.1038/srep17939

**Published:** 2015-12-14

**Authors:** Xueqiang Zhao, Jing Kong, Yuxia Zhao, Xuping Wang, Peili Bu, Cheng Zhang, Yun Zhang

**Affiliations:** 1 The Key Laboratory of Cardiovascular Remodeling and Function Research, Chinese Ministry of Education and Chinese Ministry of Health, and the State-Province Co-cultivated Key Laboratory of Translational Cardiovascular Medicine, Qilu Hospital of Shandong University, Jinan, Shandong 250012, China; 2 Department of Cardiology, Qianfoshan Hospital of Shandong Province, Jinan, Shandong 250014, China; 3 Department of Traditional Chinese Medicine, Qilu Hospital of Shandong University, Jinan, Shandong 250012, China

## Abstract

We aimed to test the hypothesis that gene silencing of tumor necrosis factor alpha converting enzyme (TACE) may attenuate lesion inflammation and positive vascular remodeling and enhance plaque stability in a rabbit model of atherosclerosis. Lentivirus-mediated TACE shRNA was injected into the abdominal aortic plaques of rabbits which effectively down-regulated TACE expression and activities from week 8 to week 16. TACE gene silencing reduced remodeling index and plaque burden, and diminished the content of macrophages and lipids while increased that of smooth muscle cells and collagen in the aortic plaques. In addition, TACE gene silencing attenuated the local expression of P65, iNOS, ICAM-1, VEGF and Flt-1 and activities of MMP9 and MMP2 while increased the local expression of TGF-β1 together with reduced number of neovessels in the aorta. TACE shRNA treatment resulted in down-regulated expression of TACE in macrophages and blunted ERK-P38 phosphorylation and tube formation of co-cultured mouse vascular smooth muscle cells or human umbilical vein endothelial cells. In conclusion, gene silencing of TACE enhanced plaque stability and improved vascular positive remodeling. The mechanisms may involve attenuated local inflammation, neovascularization and MMP activation, as well as enhanced collagen production probably via down-regulated ERK-NF-κB and up-regulated TGF-β1 signaling pathways.

Tumor necrosis factor alpha converting enzyme (TACE), also known as ADAM17 (A disintegrin and A metalloproteinase 17), was initially discovered as a protease that cleaves the 26-kDa precursor of TNF-α and sheds transmembrane TNF-α to generate a soluble form of TNF-α that can bind to TNF-α receptors to induce inflammatory response^1^. Recently it has been recognized that TACE is a type I transmembrane protein and a member of a superfamily of Zn dependent metalloproteases. The major physiological role of TACE is to regulate the proteolytic release of a number of growth factors, cytokines, adhesion molecules and cleavage enzymes from cellular membrane^2^,^3^. The major pro-inflammatory cytokine processed by TACE is TNF-α which is produced by macrophages, monocytes and T-cells, and acts as a major player in the pathogenesis of inflammation. It has been increasingly recognized that TACE-mediated shedding is involved in a variety of diseases such as ischemia, heart failure, arthritis, atherosclerosis, diabetes, cancer, neurological and immune diseases^3^,^4^,^5^. Ashley EA *et al*. provided functional evidence for TACE as a candidate gene of atherosclerosis susceptibility and found that polymorphisms of the TACE genes had relation to cardiovascular mortality in humans^6^. Canault M *et al*. found that TACE expression was associated with lesion formation in atherosclerosis-prone sites of apolipoprotein E-knockout (ApoE^-/-^) mice^7^. Oksala N demonstrated that TACE was upregulated in advanced human atherosclerotic lesions in samples from carotid, aortic, and femoral arteries compared to samples from internal thoracic artery free of atherosclerotic plaques^8^. In addition, Stoehr R *et al*. showed that loss of TIMP3, an endogenous inhibitor of TACE, exacerbated atherosclerosis in part due to unrestrained TACE activity^9^. These studies suggested that TACE is likely involved in the process of atherogenesis. However, the causative role of TACE in the formation and progression of atherosclerosis remains an open question.

Pathological studies have demonstrated that in the majority of patients with acute coronary syndromes, the culprit lesions are caused by intraluminal thrombosis secondary to rupture of vulnerable plaques, which are characterized by thinning of the fibrous cap, scarcity of collagen and smooth muscle cells, abundance of macrophages and activated T cells, and intraplaque neovascularization^10^,^11^. Although a variety of mechanisms have been proposed, overwhelming evidence suggests that local inflammation plays a major role in initiating the cascade of plaque instability^12^. Macrophages abundant in vulnerable plaque excrete a variety of cytokines and matrix-metalloproteinases (MMPs), which may impede the synthesis or accelerate the degradation of collagen in the fibrous cap, leading to so called thin-cap fibroatheroma. Our previous study *in vitro* demonstrated that TNF-α suppresses collagen production by specifically inhibiting P4Hα1, a rate-limiting enzyme for collagen synthesis, via a novel ASK1-JNK-NonO pathway^13^. However, the effect of TACE on collagen production and degradation in atherosclerotic plaques is still unknown. In the present study, we hypothesized that TACE may promote plaque instability by enhancing inflammatory response in atherosclerotic lesions and gene silencing of TACE may attenuate lesion inflammation and positive vascular remodeling and thus enhance plaque stability. A series of *in vivo* and *in vitro* experiments were designed and performed to test this hypothesis and the possible mechanisms underlying these effects were investigated.

## Results

### TACE expression in atherosclerotic plaques

**Figure 1:**
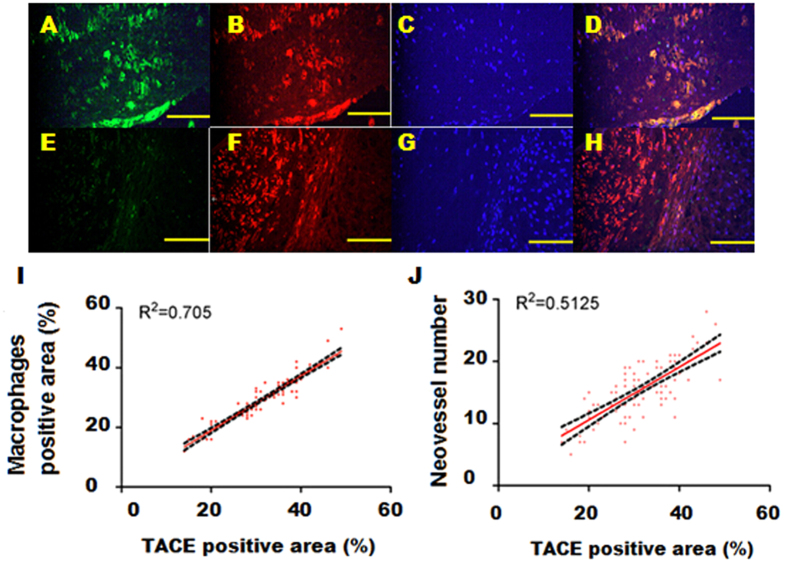
Effects of TACE shRNA treatment on TACE activity in the abdominal aortic plaques in three groups of rabbits. (**A**) Representative immnofluorescence images showing TACE positive staining cells in green; (**B**) RAM-11 positive staining cells in red; (**C**) DAPI positive staining cells in blue; (**D**) TACE, RAM-11 and DAPI positive staining cells in merged image; (**E**) TACE positive staining cells in green; (**F**) α-actin positive staining cells in red; (**G**) DAPI positive staining cells in blue; (**H**) TACE, α-actin and DAPI positive staining cells in merged image; (**I**) correlation analysis between TACE and macrophage positive staining areas; (**J**) correlation between TACE positive staining area and neovessel number in plaques. Bar = 100 μm

In the rabbit abdominal aorta, we analyzed atherosclerotic plaques from 100 sections. TACE expression was mainly observed in RAM-11-positive areas of atherosclerotic lesions (Fig. 1A–D). In addition, TACE was also expressed in the intimal smooth muscle cells (Fig. 1E–H).

The expression levels of TACE in unstable plaques were significantly higher than in stable plaques (42.6 ± 7.6 *vs*. 25.2 ± 6.5%, *P* < 0.01). Moreover, TACE expression level was positively correlated with neovessel number (Fig. 1J) and macrophage content in plaques (Fig. 1I).

### TACE shRNA down-regulated TACE and MMP2 expression *in vitro*

To evaluate the efficiency of gene silencing *in vitro*, mRNA and protein expression of TACE in macrophages from rabbits was examined and found to be substantially reduced by TACE shRNA (Supplementary Fig. 1A–C). Moreover, the mRNA expression level of MMP2 was also significantly decreased after gene silencing of TACE (Supplementary Fig. 1D).

### TACE shRNA down-regulated TACE expression *in vivo*

The intra-plaque injection method has proven an effective approach to transferring gene into atherosclerotic plaques^14^,^15^. To examine the efficacy of lentivirus-mediated gene silencing *in vivo*, the levels of mRNA and protein expression of TACE in the abdominal aortic plaques was detected by RT-PCR and immunohistochemistry. Compared with the control group, the TACE protein expression levels in the TACE shRNA group were lowered by 53.5%, 70.4% and 65.1% at 1 week, 2 weeks and 8 weeks after lentivirus transfection, respectively (all *P* < 0.05, Supplementary Fig. 2A, 2C) and the TACE mRNA expression level in the TACE shRNA group was reduced by 61.3%, 76.9% and 70.1% at 1 week, 2 weeks and 8 weeks after transfection, respectively (all *P* < 0.05, Supplementary Fig. 2B). In contrast, there was no significant difference in the TACE mRNA or protein expression levels between the control and mock groups (Supplementary Fig. 2A–C). Moreover, activities of TACE were significantly lower in the TACE shRNA group than the control and mock groups (Supplementary Fig. 2D).

### TACE shRNA ameliorated abdominal aortic positive remodeling

**Figure 2:**
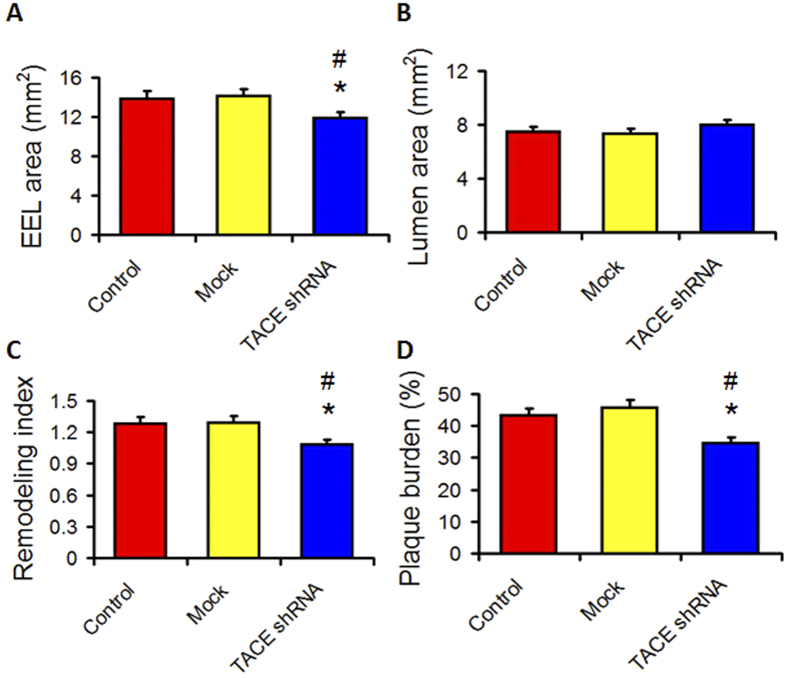
Effects of TACE gene silencing on IVUS parameters in three groups of rabbits. (**A**) External elastic lamina (EEL) area in three groups of rabbits; (**B**) Lumen area in three groups of rabbits; (**C**) Remodeling index in three groups of rabbits; (**D**) Plaque burden in three groups of rabbits; *P < 0.05 vs. Control group, ^#^*P* < 0.05 vs. Mock group.

IVUS study showed that the external elastic lamina (EEL) area was significantly smaller in the TACE shRNA group than the control and mock groups, whereas lumen area (LA) was substantially higher in the TACE shRNA group than the control and mock groups (Fig. 2A,B). Moreover, the remodeling index and plaque burden were significantly lower in the TACE shRNA group than the control and mock groups (Fig. 2C,D). However, these imaging parameters did not differ between the control and the mock groups (Fig. 2A–D).

### TACE shRNA enhanced stability of aortic plaques

**Figure 3:**
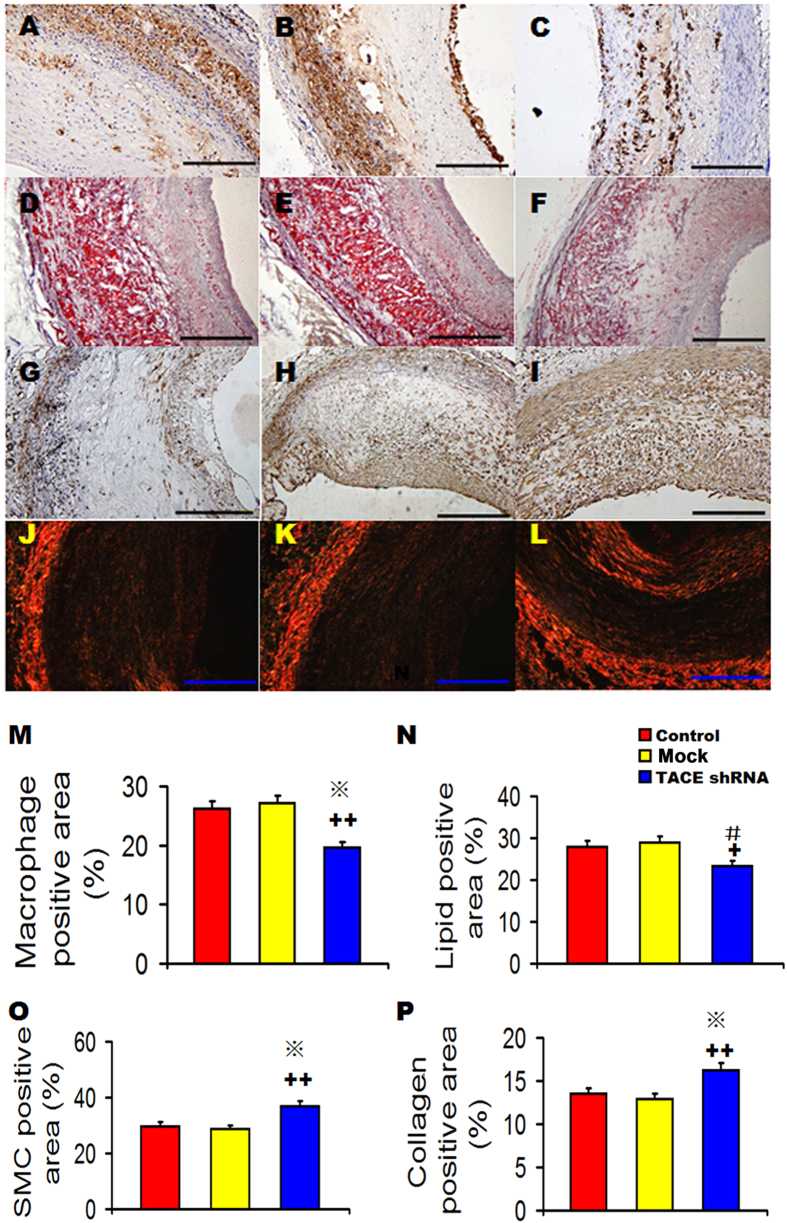
Effects of TACE gene silencing on plaque components in three groups of rabbits. (**A–C**) Representative RAM-11 immunostaining showing the macrophage content in the Control group (**A**), Mock group (**B**), and TACE shRNA group (**C**) at week 16; (**D**–**F**) Representative Oil red O staining showing the lipid content in the Control group (**D**), Mock group (**E**), and TACE shRNA group (**F**) at week 16; (**G**–**I**) Representative α-actin immunostaining showing the content of smooth muscle cells in the Control group (**G**), Mock group (**H**), and TACE shRNA group (**I**) at week 16; (**J**–**L**) Representative Sirius red staining showing the collagen content in the Control group (**J**), Mock group (**K**), and TACE shRNA group (**L**) at week 16; (**M**) Quantitative analysis of (**A**–**C**); (N) Quantitative analysis of (**D**–**F**); (**O**) Quantitative analysis of (**G**–**I**); (**P**) Quantitative analysis of (**J**–**L**). ^+^*P* < 0.05 *vs*. Control group, ^+ +^*P* < 0.01 *vs*. Control group, ^#^*P* < 0.05 *vs*. Mock group, *P < 0.01 *vs*. Mock group. Bar = 250 μm.

The relative content of macrophages and lipids in the aortic plaques was significantly lower in the TACE shRNA group than the control and mock groups (Fig. 3A–F,M,N), but did not differ between the control and the mock groups. In contrast, the relative content of SMCs and collagen in the aortic plaques of TACE shRNA group was significantly higher in the TACE shRNA group than the control and mock groups (Fig. 3G–L,O,P), but was not significantly different between the mock group and the control group. Consequently, plaque vulnerability index was significantly lower in the TACE shRNA group (0.75 ± 0.04) than the control (1.24 ± 0.03) and mock (1.23 ± 0.06, both *P* < 0.05) groups (Supplementary Fig. 3A), although it did not differ between the control and the mock groups. Correlation analysis showed that TACE activity was highly correlated with plaque vulnerability index (r^2^ = 0.9202, *P* < 0.0001, Supplementary Fig. 3B).

### TACE shRNA attenuated inflammation of aortic plaques

The protein expression levels of P65, iNOS and ICAM-1 were significantly lower in the TACE shRNA group than the control group and mock group (all *P* < 0.05), whereas the TGF-β1 expression level was significantly higher in the TACE shRNA group than the control and mock groups (Supplementary Fig. 4). However, there were no significant difference in these cytokine expression levels between the control and mock groups (Supplementary Fig. 4).

### TACE shRNA reduced MMP activities in aortic plaques

**Figure 4:**
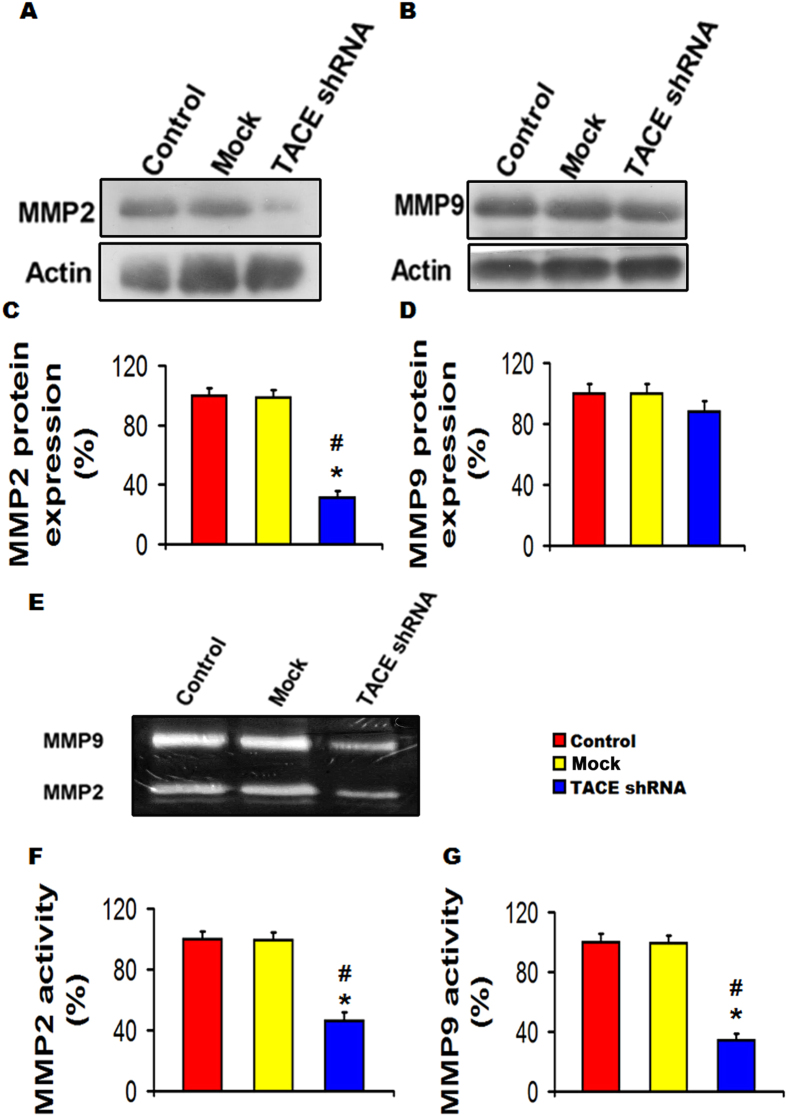
Effects of TACE gene silencing on MMP2 and MMP9 expression and activity in the abdominal aortic plaques of three groups of rabbits. (**A**) Representative western blot showing of MMP2 expression; (**B**) Representative western blot showing of MMP9 expression; (**C**) Quantitative analysis of (**A**); (**D**) Quantitative analysis of (**B**); (**E**) Representative gelatin zymography showing MMP2 and MMP9 activities; (**F**) and (**G**) Quantitative analysis of (**E**). *P < 0.01 *vs*. Control group, ^#^*P* < 0.01 *vs*. Mock group. All the gels have been run under the same experimental conditions. Black lines indicated the cropped gels and blots, and full-length blots/gels were presented in Supplementary Fig. 6.

Western-blot showed that the protein expression level of MMP2 but not MMP9 was substantially lower in the TACE shRNA group than the control group and mock groups (Fig. 4A–D). However, gelatin zymography revealed both activities of MMP9 and MMP2 were dramatically down-regulated in the TACE shRNA group than the control and mock groups (Fig. 4E–G).

### TACE shRNA diminished neovascularization of aortic plaques

**Figure 5:**
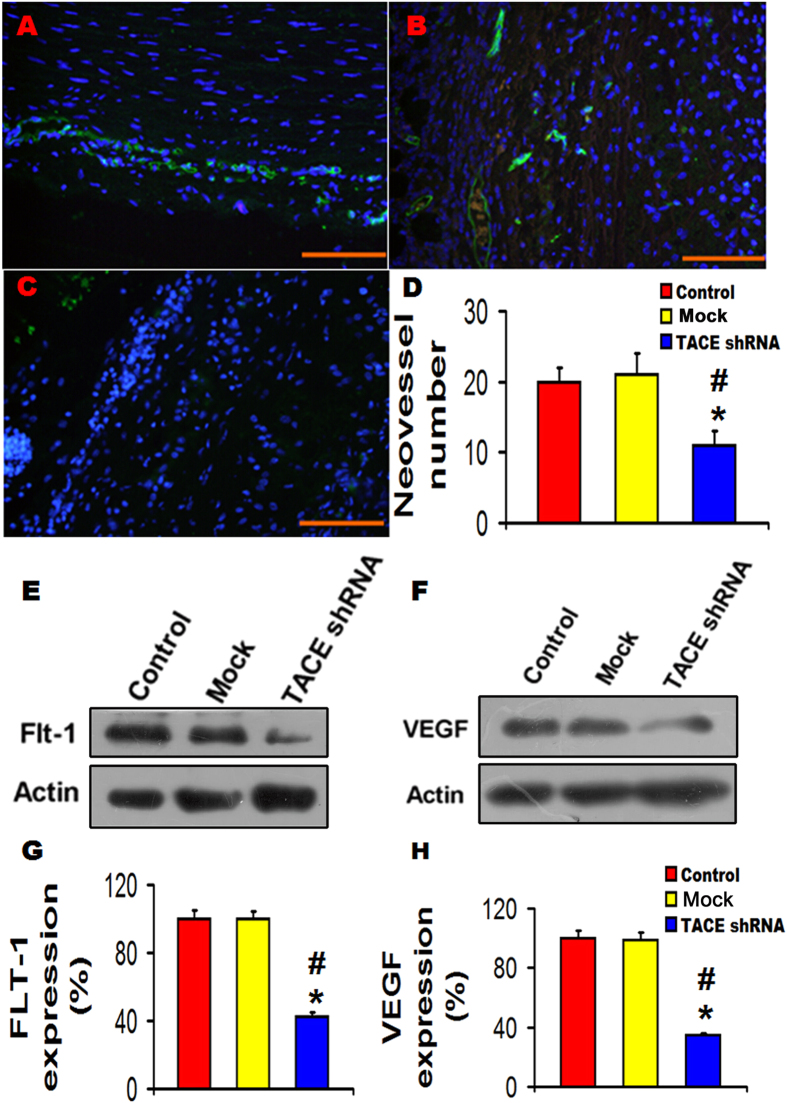
Effects of TACE gene silencing on neovascularization in the abdominal aortic plaques of three groups of rabbits. (**A**–**C**) Representative immnofluorescence images showing CD31-marked neovessels in the Control group (**A**), Mock group (**B**) and TACE shRNA group (**C**); (**D**) Quantitative analysis of neovessel number of (**A**–**C**); (**E–F**) Representative western blots showing expression of Flt-1 and VEGF in three groups of rabbits; (**G**) Quantitative analysis of Flt-1 expression in (**E**); (**H**) Quantitative analysis of VEGF expression in (**F**). *P < 0.01 *vs*. Control group, ^#^*P* < 0.01 *vs*. Mock group. Bar = 100 μm. All the gels have been run under the same experimental conditions. Black lines indicated the cropped blots, and full-length blots were presented in Supplementary Fig. 6.

The number of neovessels was significantly decreased in the TACE shRNA group in comparison with the control and the mock groups (Fig. 5A–D). Moreover, both protein expression levels of Flt-1 and VEGF were significantly lower in the TACE shRNA group than those of the control group and the mock group (Fig. 5E–H). However, there was no significant difference between the control and the mock groups in neovessel number, and Flt-1 and VEGF protein expression (Fig. 5).

### Serum lipid levels and soluble inflammatory factors

As shown in Table 1, the serum level of total cholesterol, low density lipoprotein cholesterol, high density lipoprotein cholesterol and body weight did not differ among the three rabbit groups (*P* > 0.05), suggesting that the therapeutic effects of TACE gene silencing were independent of serum lipid levels.

The serum levels of soluble inflammatory factors including sTNF-α, sICAM-1, sVCAM-1 and sMCP-1 were significantly lower in the TACE shRNA group than the control and the mock groups, and the relative reduction of sTNF-α, sICAM-1, sVCAM-1 and sMCP-1 in the TACE shRNA group in comparison to the control and the mock groups was 72%, 26%, 28% and 21%, respectively (Table 1). However, these differences were not significant between the control and the mock groups.

Correlation analysis showed that the serum concentration of sTNF-α was positively correlated with vulnerability index (r^2^ = 0.6657, *P* < 0.05, Supplementary Fig. 3C), but this correlation coefficient was less than that between TACE activity and vulnerability index (Supplementary Fig. 3B). Multiple linear regression analysis was used to assess the association between vulnerability index and soluble inflammatory factors including sTNF-α, sICAM-1, sVACM-1 and sMCP-1, and the result showed that vulnerability index was independently correlated with sTNF-α (β = 0.591, *P* < 0.001) and sICAM-1 (β = 0.363, *P* < 0.001), indicating that sTNF-α and sICAM-1 played major roles in plaque vulnerability (Table 2).

### TACE siRNA blunted ERK phosphorylation and tube formation but increased collagen production *in vitro*

**Figure 6:**
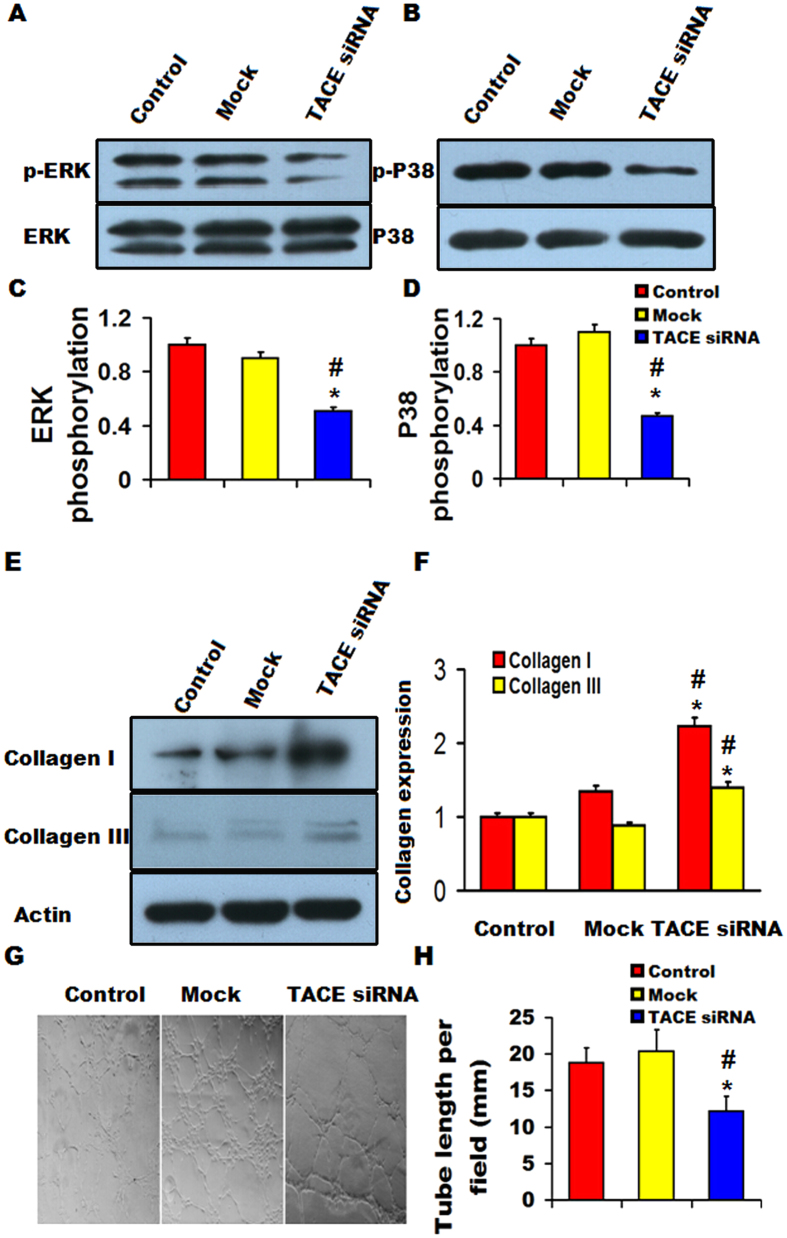
Effects of TACE gene silencing on of ERK and P38 phosphorylation, collagen production and tube formation *in vitro*. (**A**) Representative western blots showing ERK phosphorylations in mouse VSMCs; (**B**) Representative western blots showing P38 phosphorylations in mouse VSMCs; (**C**) Quantitative analysis of (**A**); (**D**) Quantitative analysis of (**B**); (**E**) Representative western blots showing collagen I and collagen III expression in mouse VSMCs; (**F**) Quantitative analysis of (**E**) ; (**G**) neovessel tube formation in three groups of HUVECs; (**H**) Quantitative analysis of (**G**). *P < 0.05 *vs*. Control group, ^▲^*P* < 0.01 *vs*. Mock group. All the gels have been run under the same experimental conditions. Black lines indicated the cropped blots, and full-length blots were presented in Supplementary Fig. 6.

After mouse VSMCs were co-cultured with RAW 264.7 cells transfected with TACE siRNA for 24 h, ERK and P38 phosphorylation was significantly decreased while collagen I and collagen III production substantially increased in mouse VSMCs relative to the control and mock groups (Fig. 6A–F). Furthermore, after HUVECs were co-cultured with THP-1 cells transfected with TACE siRNA for 24h, tube formation from HUVECs was substantially attenuated in comparison with the control and mock groups (Fig. 6G–H). The potential signaling pathways involved in the therapeutic effects of TACE gene silencing were summarized in Supplementary Fig. 5.

## Discussion

TACE, also called ADAM17, has been found to play an important role in the pathogenesis of inflammatory releases and atherosclerosis. However, the causative role of TACE in plaque stability remains obscure. In the current study, we firstly reported that endogenous TACE was highly expressed in unstable plaques and gene silencing of TACE enhanced plaque stability and improved vascular positive remodeling. The mechanisms mediating these salutary effects may involve attenuated macrophage infiltration, inflammatory cytokine expression, neovascularization and MMP2 and MMP9 activation, as well as enhanced collagen production in atherosclerotic plaques probably via down-regulated ERK- NF-κB signaling pathway and up-regulated TGF-β1 signaling.

In the majority of patients with acute coronary syndromes, the adverse cardiovascular events are caused by rupture of vulnerable plaques^16^. Pathologically, vulnerable plaques are characterized by a large necrotic core, a thin fibrous cap, abundant macrophages and scarce collagen and SMCs^17^. Although an ideal animal model of unstable plaques has yet to be established, rupture-prone plaques in NZW rabbits receiving aortic balloon injury and a high cholesterol diet exhibited many human pathological features of unstable plaques as shown by our previous studies^14^,^18^. In several previously published reports, a fibrous cap thickness less than 100 μm has been identified as a marker of unstable plaques^19^,^20^. Thus, we used this cut-off value to differentiate unstable from stable plaques in the present study. As expected, TACE expression was found to be significantly higher in unstable than stable plaques, and positive TACE staining was located mainly in macrophages and intimal SMCs. Moreover, TACE expression in aortic plaques correlated significantly with macrophage content and neovessel number in plaques. These results suggest that endogenous TACE may play an important role in the progression and instability of atherosclerotic plaques. On the other hand, gene silencing of TACE in our rabbit model significantly reduced macrophages and lipid content while increased collagen content and SMCs in atherosclerotic plaques. As a consequence, plaque vulnerability index, a pathological parameter reflective of the likelihood of a given plaque to develop rupture, was greatly diminished by TACE gene silencing. These results demonstrated for the first time that TACE has a causative role in the pathogenesis of plaque instability. Moreover, the remodeling index and plaque burden in the aortic plaques were also substantially decreased in the TACE shRNA group, suggesting that TACE gene silencing is capable of not only stabilizing but also attenuating atherosclerotic plaques.

An important feature of vulnerable plaques is a lack of sufficient collagen fibers in the fibrous cap to endure the mechanical stress imposed by pulsatile blood flow, which have been attributed to decreased collagen synthesis and increased collagen degradation in plaques. Our previous study found that TNF-α directly inhibited P4Hα1, a rate-limiting enzyme for collagen synthesis, via ASK1-JNK-NonO pathway^13^, which may partially explain why gene silencing of TACE remarkably increased collagen production in the aortic plaques. In addition, increased MMP activity in plaques appears to be the most important risk factor of plaque rupture. Both MMP9 and MMP2 localized in human vulnerable plaques are capable of active collagenolysis and have been reported to contribute to plaque expansion and rupture^21^,^22^. In the present study, we found that gene silencing of TACE substantially down-regulated the activities of MMP2 and MMP9, which was in accordance with a previous report that TACE may promote the transcription of MMP2^23^. Moreover, activated TACE may drive macrophage homing by generating soluble TNF-α, while gene silencing of TACE may decrease macrophages infiltration, which may also contribute to the attenuation of MMP activities^2^. Furthermore, recent studies showed that inhibition of TACE resulted in an increased expression of TGF-β1 signaling^24^, while TGF-β1 signaling played a crucial role in collagen production. In this study, we found that protein expression of TGF-β1 was significantly up-regulated in the TACE shRNA group. These results suggest that decreased activities of MMP2 and MMP9 and increased expression of TGF-β1 are probably the two major mechanisms underlying the therapeutic effects of TACE gene silencing on collagen production in the aortic plaques observed in this study.

A wealth of evidence indicates that an intensified inflammatory response is the major mechanism responsible for plaque instability. TACE reportedly triggers the release of a large variety of substrates, including cytokines, adhesion molecules and growth factors^5^. Recently, it has been reported that angiotensin II may activate TACE which induces shedding of ACE2, a negative regulator of angiotensin II, thus leaving the pro-inflammatory effects of angiotensin II unopposed^25^. More recently, we found that C-reactive protein may activate TACE that releases soluble lectin-like oxidized low-density lipoprotein receptor-1 from macrophages into circulation^15^. These results suggest that inflammatory factors may activate TACE which in turn leads to increased release of soluble inflammatory factors, forming a vicious cycle of “inflammation begetting inflammation”. The release of adhesion molecules by TACE may stimulate adhesive leukocytes to migrate and infiltrate into the inflamed tissue^26^. In this study, we found that gene silencing of TACE markedly decreased the serum levels of sTNF-α, sICAM-1, sVCAM-1 and sMCP-1 with the most significant reduction of sTNF-α, suggesting that TNF-α is likely the most important substrate of TACE. Previous studies found that the deficiency of TNF-α in ApoE^−/−^ mice reduced the lesion size of atheroma^27^, and a recent elegant study by Federici M’s group clearly demonstrated that patients with a higher ADAM 17 score including sICAM-1 and sVCAM-1 exhibited increased cardiovascular events^28^. The present study revealed the independent role of sTNF-α and sICAM-1 in plaque vulnerability in our animal model, which lent support to Federici M’s study. In addition, we found TACE activity correlated more closely than sTNF-α with plaque vulnerability index, suggesting that the salutary effects of TACE gene silencing observed in this study were probably due to inhibition of multiple substrates of TACE.

NF-κB is known to regulate inflammatory responses by interacting with the inflammatory signaling pathways at several levels and promoting inflammatory factor expression, including iNOS and MCP-1^29^,^30^, whereas down-regulation of ERK signaling may inactivate NF-κB as demonstrated by our previous study^31^. In the present study, we found that gene silencing of TACE substantially decreased ERK and P38 phosphorylation, and P65 and iNOS expression, suggesting that the ERK-NF-κB signaling pathway was suppressed by TACE shRNA treatment. Thus, the powerful inhibition of local inflammation may be a major contributor to the therapeutic effect of TACE gene silencing in our rabbit model. In addition, recent studies reported that neovascularization in the adventitia plays an important role in creating a conduit for lipids and inflammatory cells to be transported into plaques to enlarge the necrotic core and enhance plaque instability^32^,^33^. Analysis of the atherosclerotic plaques has demonstrated that areas of tissue injury are spatially associated with infiltrating macrophages, in association with neovascularization of the media and adventitia^34^. Moreover, VEGF/Flt-1 pathway is known to enhance the production of pro-inflammatory, chemotactic mediators and adhesion molecules^35^, and potentiate their functions in an autocrine or paracrine manner^36^. Our experiment *in vivo* demonstrated that the increased protein expression of VEGF and its receptor Flt-1 as well as neovascularization in atherosclerotic plaques were reduced by TACE gene silencing, which lent support to a recent study that pathological neovascularization was attenuated by inactivation of TACE in endothelial cells^36^,^37^. Our experiment *in vitro* also revealed that after HUVECs co-cultured with TACE downregulated THP-1 cells, neovessel tubes derived from HUVECs were dramatically decreased. These findings suggest that activated TACE and neovascularization contributed synergistically to the development of vulnerable plaques by increasing chemotaxis and transportation of inflammatory cells. Thus, attenuated neovascularization by TACE gene silencing also contributed considerably to the stabilization of atherosclerotic plaques. It has been claimed that TACE is unlikely involved in normal developmental angiogenesis, and thus TACE may offer a promising target for the treatment of pathological neovascularization^23^.

## Conclusions

In conclusion, endogenous TACE was highly expressed in unstable plaques and gene silencing of TACE enhanced plaque stability and improved vascular positive remodeling. The mechanisms may involve attenuated macrophage infiltration, inflammatory cytokine expression, neovascularization and MMP2 and MMP9 activation, as well as enhanced collagen production in atherosclerotic plaques probably via down-regulated ERK-NF-κB and up-regulated TGF-β1 signaling pathways. Thus, TACE inhibition may provide a promising approach to the stabilization of vulnerable plaques.

## Materials and Methods

### Small hairpin RNAs interference and lentivirus construct

We used pGLV-U6-EGFP (pGLV1-1) containing U6 expression cassette, an RNA polymerase III-dependent transcription of shRNA transcript. This vector also expressed green fluorescent protein from a cytomegalovirus promoter, which allowed for monitoring of the transfection efficiency. Small hairpin RNAs were designed to contain 21-nucleotide sense sequences identical to the target molecule(s), followed by a short (7-nucleotide) nonspecific loop sequence and an antisense sequence, followed by two thymidines, which served as a stop signal for RNA polymerase III. The TACE target sequence consisted of 4 duplex sequences of target-specific 21nt siRNAs including: 5′-atagagccactttggagattt-3′, 5′-ggatttaaaggttatggaata-3′, 5′-ggacttcttcagtggacatgt-3′, 5′- ggaacacttcatgggacaatg -3′ in TACE splice variant. As a control, we used 21-nucleotide scrambled small hairpin RNA, which did not give more than an 18-nucleotide match against any rabbit genomic sequence. The oligonucleotides were annealed and cloned into pGLV1-1 between the HpaI and XhoI sites. The vector was transfected into 293T cells together with the viral packaging vectors by GenePhama, Shanghai, China.

### Animal model and TACE shRNA transfection *in vivo*

Fourty-five adult male New Zealand white (NZW) rabbits (1.9~2.1 kg in weight) received balloon induced abdominal aorta endothelium injury (3.5 mm; three times, 14–16 atm inflations) under general anesthesia with 3% pentobarbital sodium (30mg/kg) and then were fed with 1% cholesterol diet for 16 weeks. These rabbits were randomly divided into 3 groups: TACE shRNA group (n = 15, receiving 0.2 mL of 2 × 10^9^ pfu/mL recombinant Lentivirus-TACE shRNA), mock group (n = 15, receiving 0.2 mL of 2 × 109 pfu/mL recombinant Lentivirus-scramble shRNA), and control group (n = 15, receiving 0.2 mL PBS).. Blood samples of the three groups were collected at week 16 and plasma was stored at −80 °C.

In addition, 10 adult male NZW rabbits (1.9~2.1 kg in weight) received balloon induced abdominal aorta endothelium injury and were fed with 1% cholesterol diet for 16 weeks. The abdominal aortic cross sections from these 10 rabbits were used for plaques differentiation. The experiment protocol complied with the Animal Management Rule of the Ministry of Health, People’s Republic of China (documentation 55, 2001) and was approved by the Animal Care Committee of Shandong University.

### Intravascular ultrasound (IVUS) study and lentivirus suspension transduction protocol

IVUS study was performed at week 8 and 16 by use of a 3.2 F catheter containing a single rotating element transducer of 40 MHz connected to an IVUS system (Galaxy, Boston Scientific Corp, Boston, MA, USA) as reported previously^38^. IVUS study was performed according to the standard procedure. The image resolution was 150 mm and 300 mm respectively in axial and lateral direction. After the catheter advanced from the femoral artery to the level of the left renal artery, it was withdrawn by use of a motorized pullback device at a constant speed of 0.5 mm·s^−1^ till the level 8 cm below the left renal artery.

At week 8, lentivirus and PBS were transfected using an intraplaque injection method as reported previously by our laboratory^14^,^18^. Briefly, after imaging the largest plaque in the abdominal aorta by IVUS, different lentivirus suspension or PBS was injected into the corresponding portion of the abdominal aorta with the guidance of IVUS, respectively, and the injected site was marked by an iliopsoas stitch. Then we closed the abdominal cavity and gave animals antibiotics intramuscularly to prevent infection.

At week 16, IVUS was repeated. External elastic lamina (EEL) area and lumen area were measured from cross-sectional images and remodeling index (RI) was calculated as the ratio of EEL area of the lesion segment to the EEL area of the reference segment. Plaque burden was calculated as (EEL area-lumen area)/ EEL area × 100%. The segments without visible lesion at a distance of 10-mm from the plaque were regarded as the reference segments, and the mean EEL area derived from 5 different reference segments or lesion segments was calculated. Plaques with RI > 1.05 was regarded as positive remodeling, RI = 0.95–1.05 as no remodeling, and RI  < 0.95 as negative remodeling. The IVUS images were reviewed by two independent observers and the averaged values were used for data analyses.

### Histopathological analysis

After IVUS study at week 16, animals were administered an overdose of sodium pentobarbital followed by intravenous injection with heparin (500 U/kg). Thereafter, the abdominal aortic segments from the level of the left renal artery to the level of 8 cm below the left renal artery was excised, rinsed with PBS and cut in cross sections at 3 to 6 mm intervals. Some nonadjacent sections of each aortic plaque were post-fixed in 4% buffered paraformaldehyde and embedded in paraffin or OCT (frozen tissue matrix) for frozen sections, while other sections were stored at −80 °C. Morphological changes of the plaques and aortic walls were observed in sections stained with HE staining, Sirius red staning, oil red O staining and immunohistochemistry. To quantify lipid depositions of the abdominal aorta, 5μm sequential sections were cut from aortic segments embedded in OCT and an average of lipid deposition from nonadjacent 20 separate sections in each aortic ring was calculated. The relative positive staining area for macrophages, lipids, smooth muscle cells (SMCs) and collagen were measured by an image analysis system (Image-Pro Plus 5.0, Media Cybernetics, USA) attached to a color CCD video camera and plaque vulnerable index was calculated as: (macrophages% + lipids %)/(smooth muscle cells% + collagen%)^14^,^38^.

### Classification of atherosclerotic plaques

The atherosclerotic plaques were classified into two groups based on the fibrous cap thickness of plaques with the cut-off value of 100μm^19^: stable plaques (>100 μm) and unstable plaques (≤100 μm). Cap thickness was measured at the point of the thickest lipid core with a clear demarcation of the fibrous cap-lipid core interface. The lipid core stained with oil red O staining was defined as a layer more than 100μm thick consisting of foam cells and extracellular lipid deposits. Fibrous caps in adjacent species stained with Sirius red were defined as areas consisting of SMCs and collagen fibers covering the lipid core. Lipid cores and fibrous caps were measured with an image analysis system (Image-Pro Plus 5.0, Media Cybernetics, USA) attached to a color CCD video camera.

### Immunohistochemical analysis

Serial sections were subjected to immunostaining for RAM11 monoclonal antibody (1:200, Lab Vision Neomarkers, USA), mouse anti-human smooth muscle cell α-actin 1A4 monoclonal antibody (1:100, Boster, China), goat anti-human PECAM-1 (CD31) polyclonal antibody (1:100, Santa Cruz Biotechology, USA), goat anti-human TACE polyclonal antibody (1:100, Santa Cruz Biotecholog, USA), mouse anti-human P65 monoclonal antibody (1:200, Santa Cruz Biotecholog, USA), goat anti-human iNOS polyclonal antibody (1:200, Santa Cruz Biotecholog, USA), goat anti-human ICAM-1 polyclonal antibody (1:200, Santa Cruz Biotecholog, USA), and goat anti-human TGFβ1 polyclonal antibody (1:200, Santa Cruz Biotecholog, USA). Briefly, sections were treated with 3% hydrogen peroxide for 10 minutes, blocked with 5% normal BSA at 37 °C for 20 minutes, and incubated with the primary antibody at 4 °C overnight. Appropriate negative controls using non-immune IgG, phosphate-buffered saline instead of the primary antibody or no primary and secondary antibodies were applied. The color was developed with the DAB chromogen and counterstained with hematoxylin.

### Quantification of Neovascularization

Neovessels were identified as channels surrounded by a layer of endothelial cells highlighted by immunostaining with anti-CD31 antibody. We calculated the adventitia neovessels in the area encircling the EEL within one × 200 optical field. All neovessels in the section were counted by two observers by use of fields at × 200 magnification with Image Pro-plus (Media Cybernetics, USA).

### Western Blot Analysis

Protein was extracted from tissues or cell lystes and the protein content was measured by a Bio-Rad protein assay. Equal amounts of protein (30 μg/lane) were separated by 10% SDS-PAGE and electro-transferred to nitrocellulose membranes. Nonspecific antibody binding was blocked with 5% skim milk in TBS. Membrane was incubated overnight with a primary antibody for goat anti-human TACE polyclonal antibody (1:1000, Santa Cruz Biotecholog, USA), goat anti-human MMP2 polyclonal antibody (1:1500, Santa Cruz Biotecholog, USA), goat anti-human MMP9 polyclonal antibody (1:1500, Santa Cruz Biotecholog, USA), goat anti-human VEGF polyclonal antibody (1:1000, Santa Cruz Biotecholog, USA), goat anti-human Flt-1 polyclonal antibody (1:1000, Santa Cruz Biotecholog, USA), rabbit anti-mouse collagen I polyclonal antibody (1:1000, Abcam), rabbit anti-mouse collagen III polyclonal antibody (1:5000, Abcam), rabbit anti-mouse ERK polyclonal antibody (1:1000, Cell Signal), rabbit anti-mouse p-ERK polyclonal antibody (1:1000, Cell Signal), rabbit anti-mouse P38 polyclonal antibody (1:1000, Cell Signal), rabbit anti-mouse p-P38 polyclonal antibody (1:1000, Cell Signal) and goat anti-human β-actin polyclonal antibody (1:1500, Santa Cruz Biotecholog, USA) and then with HRP-conjugated secondary antibody for one hour in TBS. After each antibody incubation, the blot was washed (three times; 5 min each) with TBS containing 0.1% Tween 20. The antigen-antibody complexes were exposed to chemiluminescent substrate (Millipore) and scanned using an image analyzer (AlphaImager 2200, Alpha). All blots were probed with β- actin as a loading control, and densitometric analysis was performed with an image analyzer (AlphaImager 2200, Alpha).

### Quantitative real-time PCR (RT-PCR)

mRNA from the abdominal aorta or macrophages of rabbit was prepared with TRIzol Reagent (Invitrogen, USA) and PrimeScript^TM^ Reverse Transcriptase (Takara Bio Inc, Japan) according to the manufacturer’s instructions. RT-PCR was used to determine the mRNA levels of TACE, MMP2 and glyceraldehyde phosphate dehydrogenase (GAPDH). The sequences of primers were shown in Supplementary Table 1. Melt curve analysis was performed after each reaction to verify that primer dimers were absent. Data analysis was performed with Bio-RadiQ5 (USA) and the 2^–△△CT^ method was used to assess the relative mRNA expression level normalized in multiplex reactions using GAPDH as control.

### Fluorescence enzymatic activity measurement

TACE activity of the abdominal aortic tissues was measured by use of the Sensolyte 520 TACE activity assay kit (Anaspec, USA) according to the manufacturer’s instruction. Briefly, after tissues were lysed in assay buffer, incubated for 10 min at 4 °C and centrifuged at 2500 g for 10 min at 4 °C, the supernatants were collected. Equal amounts of proteins were incubated with 50μl TACE substrate for 30 min at 37 °C and changes in fluorescence were monitored by Varioskan Flash software (USA) with excitation 490 nm and emission 520 nm.

### ELISA and biochemical assay

Soluble inflammatory factors of the plasma were measured by ELISA assay and biochemical assay was conducted in accordance with the manufacturer’s instruction.

### Zymography

MMP activities of the abdominal aortic plaques were assessed with zymography. Briefly, samples were minced and homogenized in ice-cold 10 mM PBS (pH 7.2) containing 150 mmol/L NaCl, 1% Triton X-100, 0.1% SDS, 0.5% sodium deoxycholate and 0.2% NaN_3_. Tissue homogenates were centrifuged at 14 000 rpm for 10 minutes at 4 °C and the supernatant was collected. Protein content was measured by a Bio-Rad protein assay, and SDS–polyacrylamide gel electrophoresis zymography was performed. One part of homogenate containing 30 μg of protein mixed with 1 part of 4 × SDS sample buffer containing 0.32% Tris-HCL, 4%SDS (PH7.2), 16% glycerol, bromophenol blue, and molecular weight markers were added. Each sample was loaded on a 10% SDS-polyacrylamide gel containing 0.1% gelatin.

### Rabbit macrophages culture and siRNA transfection

Peripheral blood samples were obtained from all rabbits and peripheral blood mononuclear cells (PBMC) were isolated by Ficoll density gradient centrifugation and cultured in RPMI 1640 medium with 20% autologous serum at 37 °C in 5% CO_2_ for 7 days to induce differentiation of PBMC into macrophages. The macrophages were washed with Opti-MEM and incubated for 2 h with Opti-MEM medium before being transfected with siRNA of TACE (50 nM) using Lipofectamine 2000 (5 μL/ml, Invitrogen) as the transfection reagent. After transfection for 12 hr, the Opti-MEM medium was replaced with fresh RPMI 1640 (containing 20% rabbits serum), and the samples were incubated for another 36 h.

### Cell culture

Mouse macrophages RAW 264.7 and vascular smooth muscle cell lines(VSMC) were cultured in Dulbecco’s Modified Eagle’s Medium(DMEM, Gibco, USA), and human monocyte cell line THP-1 was grown in RPMI1640 medium(Gibco, USA). These two media were supplemented with 10% fetal bovine serum and 1% antibiotic penicillin–streptomycin. Human umbilical vein endothelial cells (HUVECs, ATCC, USA) were maintained in ECM medium (Sciencell, USA) supplemented with fetal bovine serum, growth factors and penicillin–streptomycin, and it was passaged on marix gel-coated 24-well plates.

### Small interfering RNA (siRNA) transfection of *in vitro* experiments

RAW 264.7 and THP-1 cells were transfected with 30 nM TACE siRNA oligos (Santa Cruz Biotecholog, USA) using RNAiMAX (Invitrogen, USA) according to the manufacturer’s protocol. The transfected cells were maintained at 37 °C with 5% CO2 for 24 h.

### Cell co-culture

Mouse VSMCs were seeded at the lower chambers of a 6-well Transwell apparatus with a 0.4 μm pore-size polyester membrane (Costar, NY, USA). When VSMCs were 50% confluent, RAW 264.7 cells after TACE siRNA transfection were plated into the upper chambers of the Transwell plates. The co-culture system was maintained for 24 h at 37 °C with 5% CO_2_, and the VSMCs were harvested for protein analysis.

### Tube formation

We added 200 μl matrix gel (BD Biosciences, USA) to each lower chamber of the 24-well Transwell plate with a 0.4 μm pore-size polyester membrane (Costar, NY, USA), and the plate was incubated at 37 °C overnight to allow gel solidification. The next day, HUVEC (75000/well) were seeded on the matrix gel, and at the mean time, THP-1 cells after TACE siRNA transfection were plated into the upper chambers of the Transwell plate. The co-cultured cells were incubated at 37 °C for 6 h. The 2-dimensional organization and the network cellular growth area of each well were photographed with a Canon Act 1 phase-contrast microscope ( × 10). The formation of capillary-like tubes was captured 12 h later. The mean tube length was calculated in 5 random fields (100 × ).

### Statistical analyses

Values were expressed as mean ± SD. Statistical analysis was performed with one-way ANOVA followed by Students-Newman-Keuls (SNK) post hoc test. Correlation coefficients were assessed with Pearson correlation test. Multiple linear regression analysis was performed to evaluate the association between different variables. Statistical significance was defined as *P* < 0.05.

## Additional Information

**How to cite this article**: Zhao, X. *et al*. Gene silencing of TACE enhances plaque stability and improves vascular remodeling in a rabbit model of atherosclerosis. *Sci. Rep*. **5**, 17939; doi: 10.1038/srep17939 (2015).

## Supplementary Material

Supplementary Information

## Figures and Tables

**Table 1:** Biochemical measurements in three groups of rabbits.

	Control (n = 15)	Mock (n = 15)	TACE shRNA (n = 15)
HDL-C(mg/dl)	470.9 ± 37.7	482.6 ± 36.2	489.1 ± 38.3
LDL-C(mg/dl)	1407.6 ± 82.9	1483.6 ± 84.1	1462.1 ± 82.9
TC(mg/ml)	2050.3 ± 126.2	2096.3 ± 128.7	2074.4 ± 131.4
TG(mg/dl)	144.5 ± 10.7	148.5 ± 10.2	149.6 ± 11.0
Body weight(kg)	4.27 ± 0.53	4.33 ± 0.49	4.29 ± 0.51
sICAM-1 (pg/ml)	891.37 ± 192.18	905.74 ± 183.81	664.37 ± 141.82△▲
sVCAM-1(ng/ml)	3.39 ± 1.05	3.46 ± 1.12	2.46 ± 1.09△▲
sMCP-1 (pg/ml)	533.38 ± 130.15	548.82 ± 127.54	426.76 ± 143.68△▲
sTNF-α (pg/ml)	516.74 ± 64.73	561.38 ± 68.24	149.14 ± 17.06△▲

^△^*P* < 0.01 *vs*. Control group, ^▲^*P* < 0.01 vs. Mock group.

TC: total cholesterol; LDL-C: low density lipoprotein cholesterol; HDL-C: high density lipoprotein cholesterol; TG: triglycerides; sICAM-1: soluble intercellular cell adhesion molecule-1; sVCAM-1: soluble vascular cell adhesion molecule-1; sMCP-1: soluble monocyte chemotactic protein 1; sTNF-α: soluble tumor necrosis factor alpha.

**Table 2:** Multivariate linear correlation between soluble inflammatory factors and plaque vulnerability index.

	Correlationcoefficient	P Value	Standardizedregressioncoefficients	P Value
sTNF-α	0.816	*P* < 0.001	0.591	*P* < 0.001
sICAM-1	0.729	*P* < 0.001	0.363	*P* = 0.001
sVACM-1	0.573	*P* < 0.001	0.003	*P* = 0.981
sMCP-1	0.578	*P* < 0.001	−0.037	*P* = 0.828
