# Author Correction: Gene silencing of TACE enhances plaque stability and improves vascular remodeling in a rabbit model of atherosclerosis

**DOI:** 10.1038/s41598-022-22794-w

**Published:** 2022-11-02

**Authors:** Xueqiang Zhao, Jing Kong, Yuxia Zhao, Xuping Wang, Peili Bu, Cheng Zhang, Yun Zhang

**Affiliations:** 1 The Key Laboratory of Cardiovascular Remodeling and Function Research, Chinese Ministry of Education, Chinese Ministry of Health, The State-Province Co-Cultivated Key Laboratory of Translational Cardiovascular Medicine, Qilu Hospital of Shandong University, Jinan, 250012 Shandong China; 2 Department of Cardiology, Qianfoshan Hospital of Shandong Province, Jinan, 250014 Shandong China; 3 Department of Traditional Chinese Medicine, Qilu Hospital of Shandong University, Jinan, 250012 Shandong China

Correction to: *Scientific Reports* 10.1038/srep17939, published online 14 December 2015

This Article contains errors.

As a result of errors during figure assembly, images in panels 3E and 3K show incorrect samples. The authors were able to locate the original data, and the revised Figure 3, with corrected panels 3E and 3K, and its accompanying legend appear below.

**Figure 3:**
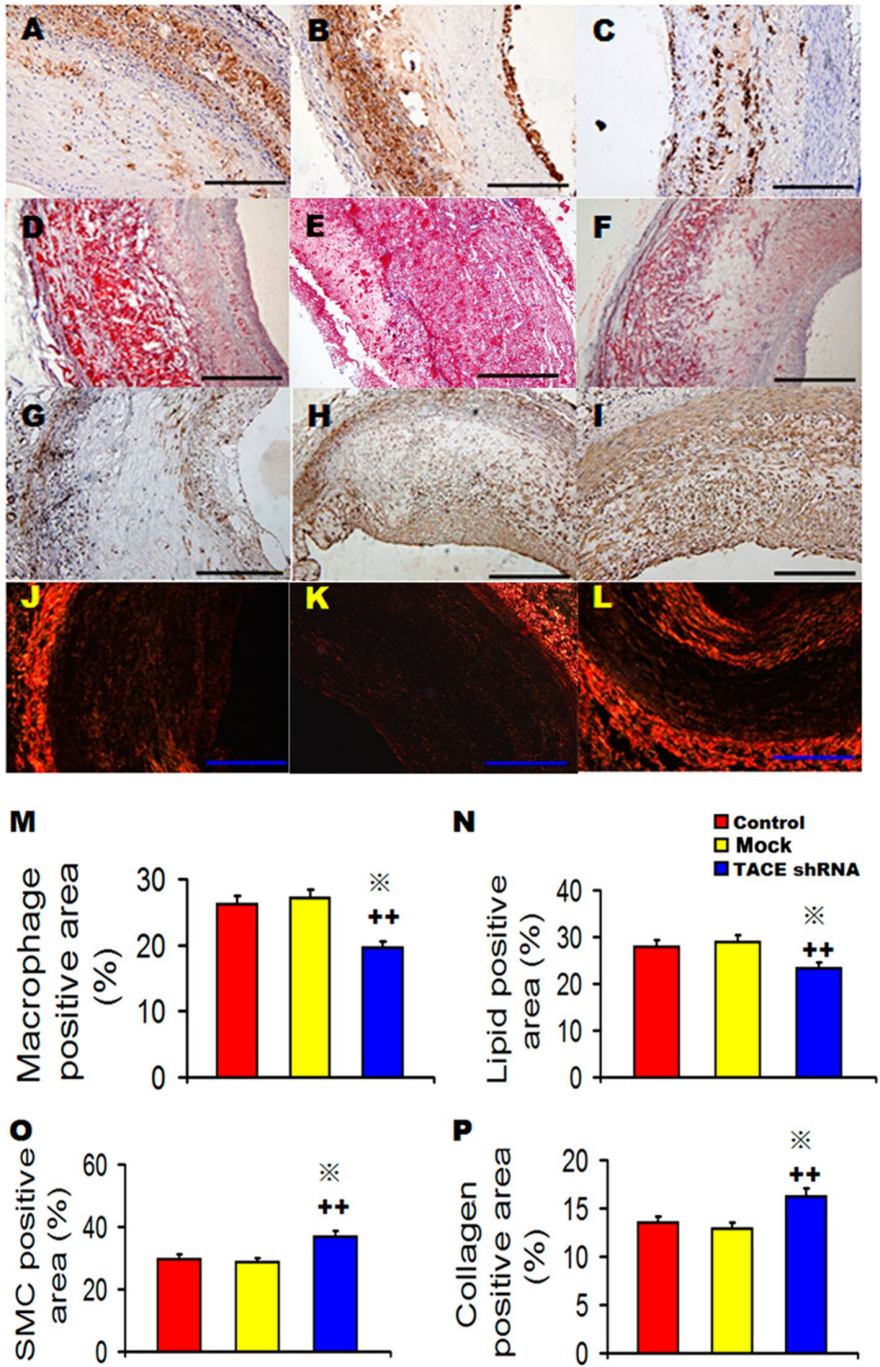
Effects of TACE gene silencing on plaque components in three groups of rabbits. (**A–C**) Representative RAM-11 immunostaining showing the macrophage content in the Control group (**A**), Mock group (**B**) and TACE shRNA group (**C**) at week 16; (**D**–**F**) Representative Oil red O staining showing the lipid content in the Control group (**D**), Mock group (**E**) and TACE shRNA group (**F**) at week 16; (**G**–**I**) Representative α-actin immunostaining showing the content of smooth muscle cells in the Control group (**G**), Mock group (**H**) and TACE shRNA group (**I**) at week 16; (**J**–**L**) Representative Sirius red staining showing the collagen content in the Control group (**J**), Mock group (**K**) and TACE shRNA group (**L**) at week 16; (**M**) Quantitative analysis of (**A**–**C**); (**N**) Quantitative analysis of (**D**–**F**); (**O**) Quantitative analysis of (**G**–**I**); (**P**) Quantitative analysis of (**J**–**L**). ^+^*P* < 0.05 vs. Control group, ^++^*P* < 0.01 vs. Control group, ^#^*P* < 0.05 vs. Mock group, *P < 0.01 vs. Mock group. Bar = 250 µm.

In addition, the Authors re-did the statistical analysis on the corrected data. As a result, the *p* value for the difference in the lipid positive area between mock and TACE shRNA groups became < 0.01 as shown in panel N of the corrected figure.

