# Supplementary material for: Gene silencing of TACE enhances plaque stability and improves vascular remodeling in a rabbit model of atherosclerosis
Source: Sci Rep. 2015 Dec 14;5:17939. doi: 10.1038/srep17939 (PMC4677302; doi:10.1038/srep17939)
Supplement: Supplementary Information [file srep17939-s1.doc]

**Supplementary data**

**Gene silencing of TACE enhances plaque stability and improves vascular remodeling in a rabbit model of atherosclerosis**

***By:***

Xueqiang Zhao ^1,2,※^, Jing Kong^1,※^, Yuxia Zhao ^3^,Xuping Wang^1^, Peili Bu ^1^, Cheng Zhang^1, *^, Yun Zhang ^1,*^

***From:***

^1^The Key Laboratory of Cardiovascular Remodeling and Function Research, Chinese Ministry of Education and Chinese Ministry of Health, and the State-Province Co-cultivated Key Laboratory of Translational Cardiovascular Medicine, Qilu Hospital of Shandong University, Jinan, Shandong 250012, China; ^2^Department of Cardiology, Qianfoshan Hospital of Shandong Province, Jinan, Shandong 250014, China;^3^Department of Traditional Chinese Medicine, Qilu Hospital of Shandong University, Jinan, Shandong 250012, China.

^※^ These authors contributed to this work equally.

**Correspondence to:** Yun Zhang, MD, PhD, FACC, FESC, FASE, [zhangyun@sdu.edu.cn](mailto:zhangyun@sdu.edu.cn) or Cheng Zhang, MD, PhD, FESC, [zhangc@sdu.edu.cn](mailto:zhangc@sdu.edu.cn), Shandong University Qilu Hospital, No.107, Wen Hua Xi Road, Jinan, Shandong, 250012, P.R.China. TEL: +86531-82169257, Fax: +86531-86169356

**Supplementary Table 1. Primer sequences**

| **Gene** | **Primer sequence** | **Accession No.** | **Product length** |
| --- | --- | --- | --- |
| **TACE** | ACTTTGGAGCAGAGCACGAT  ACCTGGGCCTTACTCTCGAT | EF472913.1 | 174bp |
| **MMP2** | TCTGCCCTGACCAAGGTTAC  CCCGTAGAGCTCTTGAATGC | NM_001082209.1 | 173bp |
| **GAPDH** | AGGTCATCCACGACCACTTC  GTGAGTTTCCCGTTCAGCTC | XM_002713034.1 | 202bp |

**Supplementary figures**

**
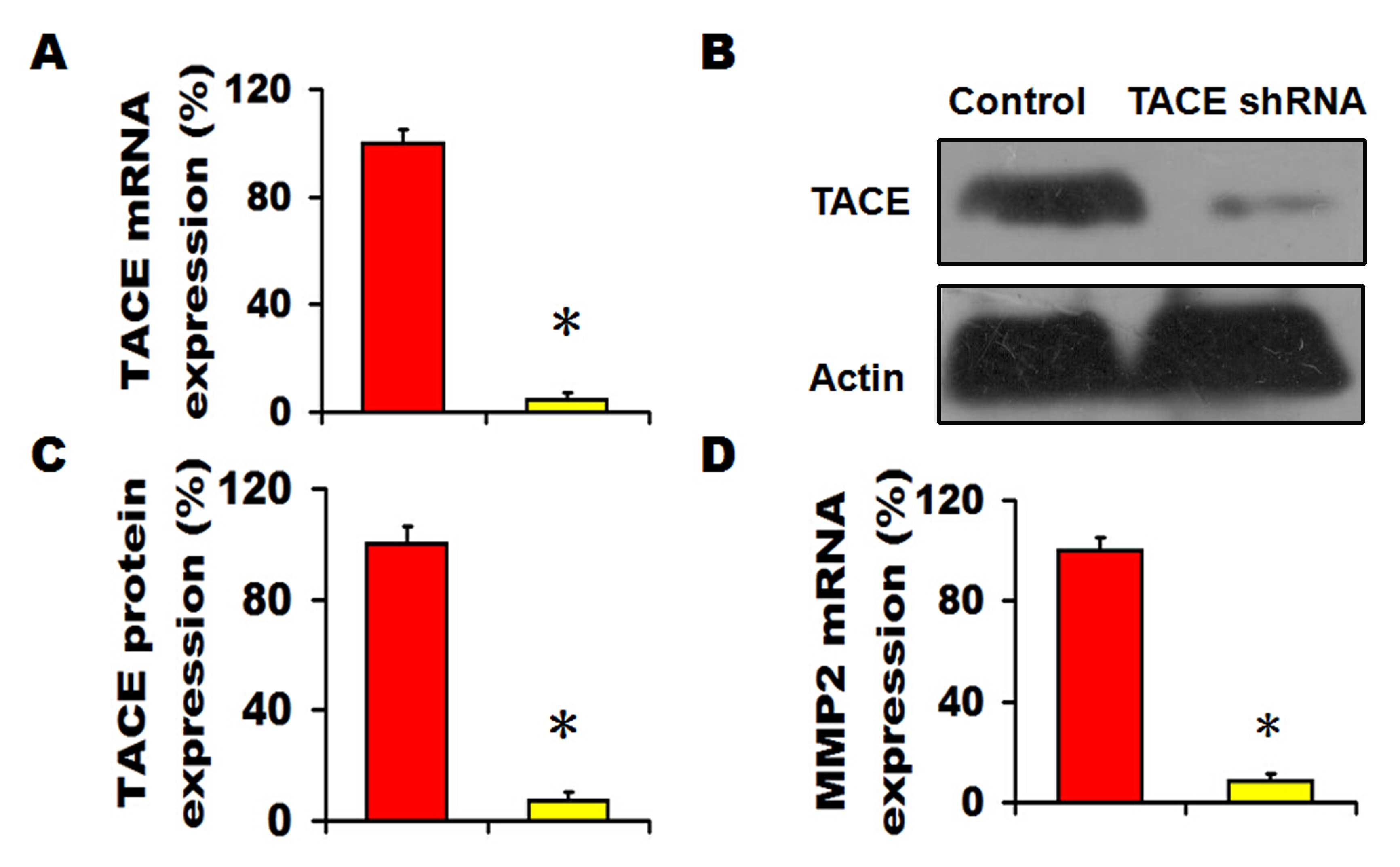
**

**Supplementary Figure 1.** **Effects of TACE shRNA treatment on mRNA and protein expression of TACE in rabbit macrophages.** (A) TACE mRNA expression levels in control and TACE shRNA groups; (B) Representative western blots showing TACE protein expression levels in control and TACE shRNA groups; (C) Quantitative analysis of (B);(D) MMP2 mRNA expression levels in control and TACE shRNA groups. ﹡*P*<0.05 *vs.* Control group. All the gels have been run under the same experimental conditions. Black lines indicated the cropped blots, and full-length blots were presented in Supplementary Figure 6.


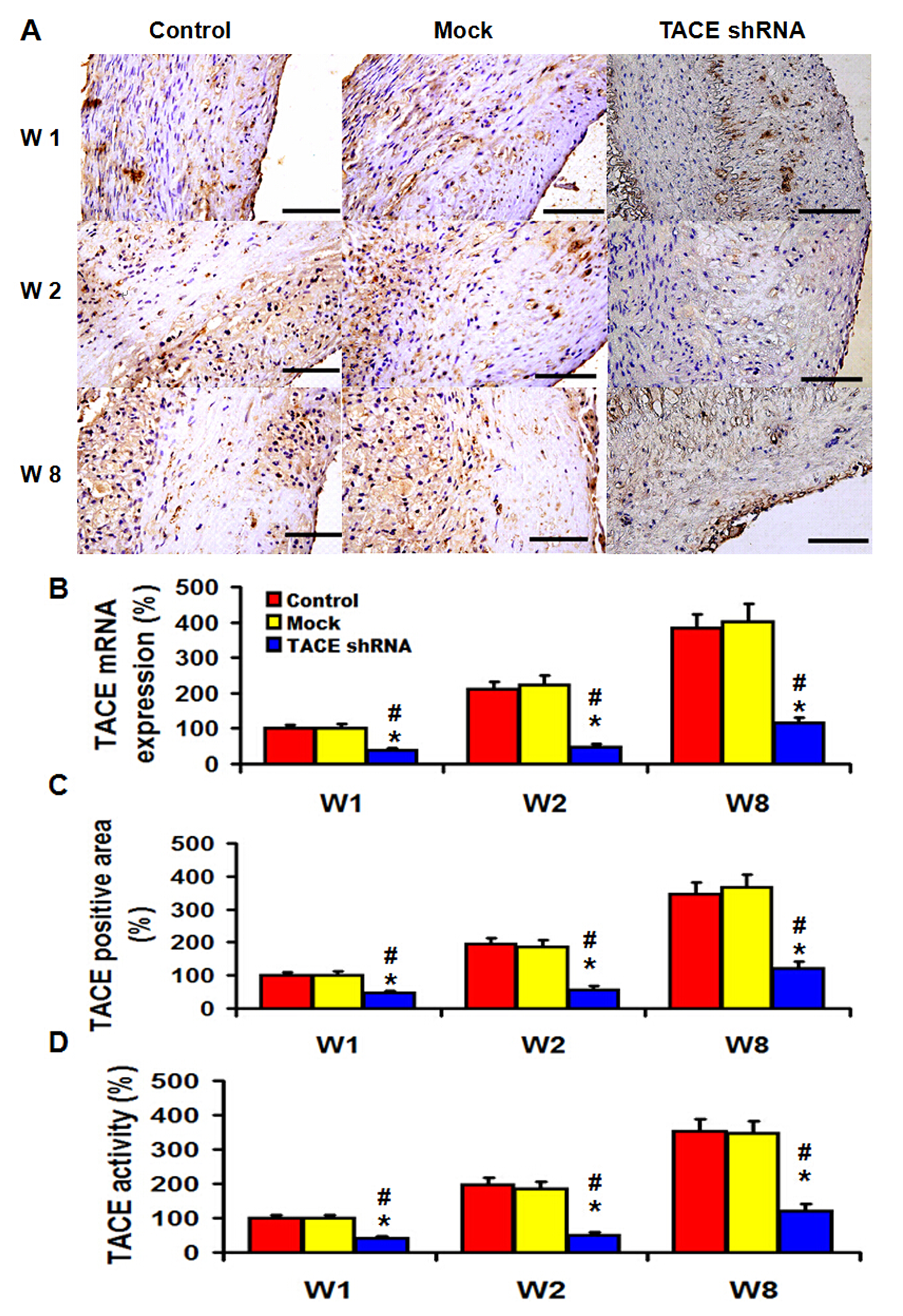


**Supplementary Figure 2.** **mRNA and protein expression levels and activity of TACE at 1 week, 2 weeks and 8 weeks after lentivirus transfection *in vivo*.** (A) Representative immunostaining images showing protein expression levels of TACE in three groups of rabbits at 1 week (W1), 2 weeks (W2) and 8 weeks (W8) after lentivirus transfection; (B) mRNA expression levels of TACE in three groups of rabbits at 1 week, 2 weeks and 8 weeks after lentivirus transfection; (C) Quantitative analysis of A; (D) Quantitative analysis of TACE activities in three groups of rabbits at 1 week, 2 weeks and 8 weeks after lentivirus transfection. ﹡*P*<0.01 *vs.* Control group, ^＃^*P* <0.01 *vs.* Mock group at the same time point. Bar=100μm


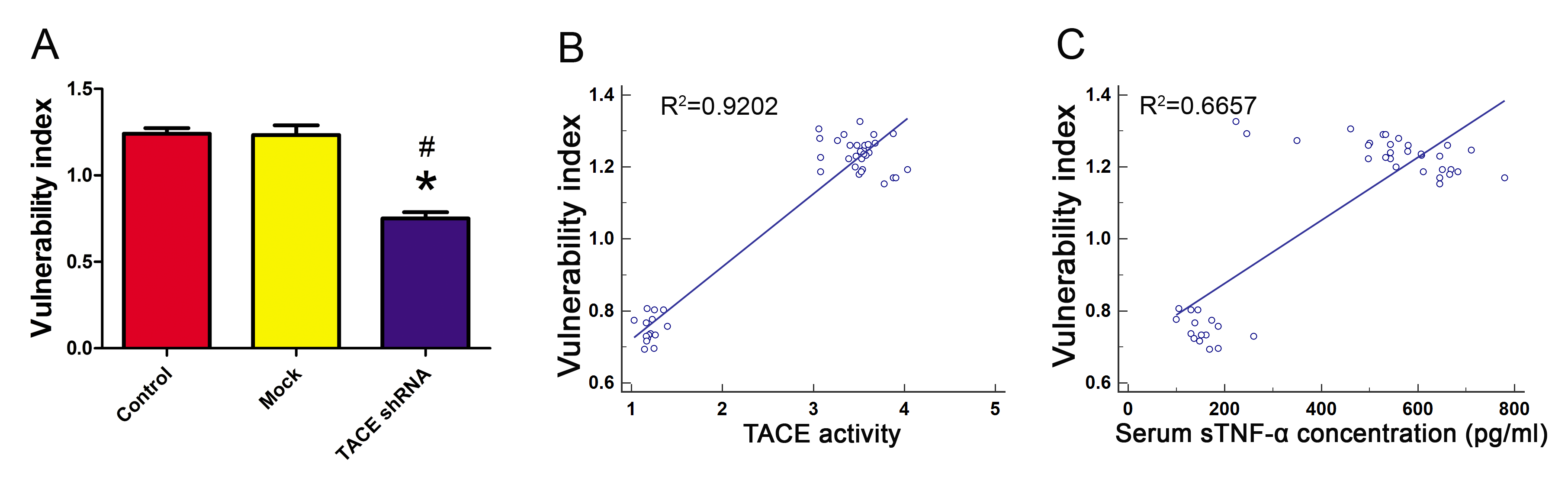


**Supplementary Figure 3. *Effects of TACE gene silencing on plaque vulnerability index.*** (A) Plaque vulnerability index in three groups of rabbits; (B) Correlation analysis between vulnerability index and TACE activity; (C) Correlation analysis between vulnerability index and serum sTNF-α level.


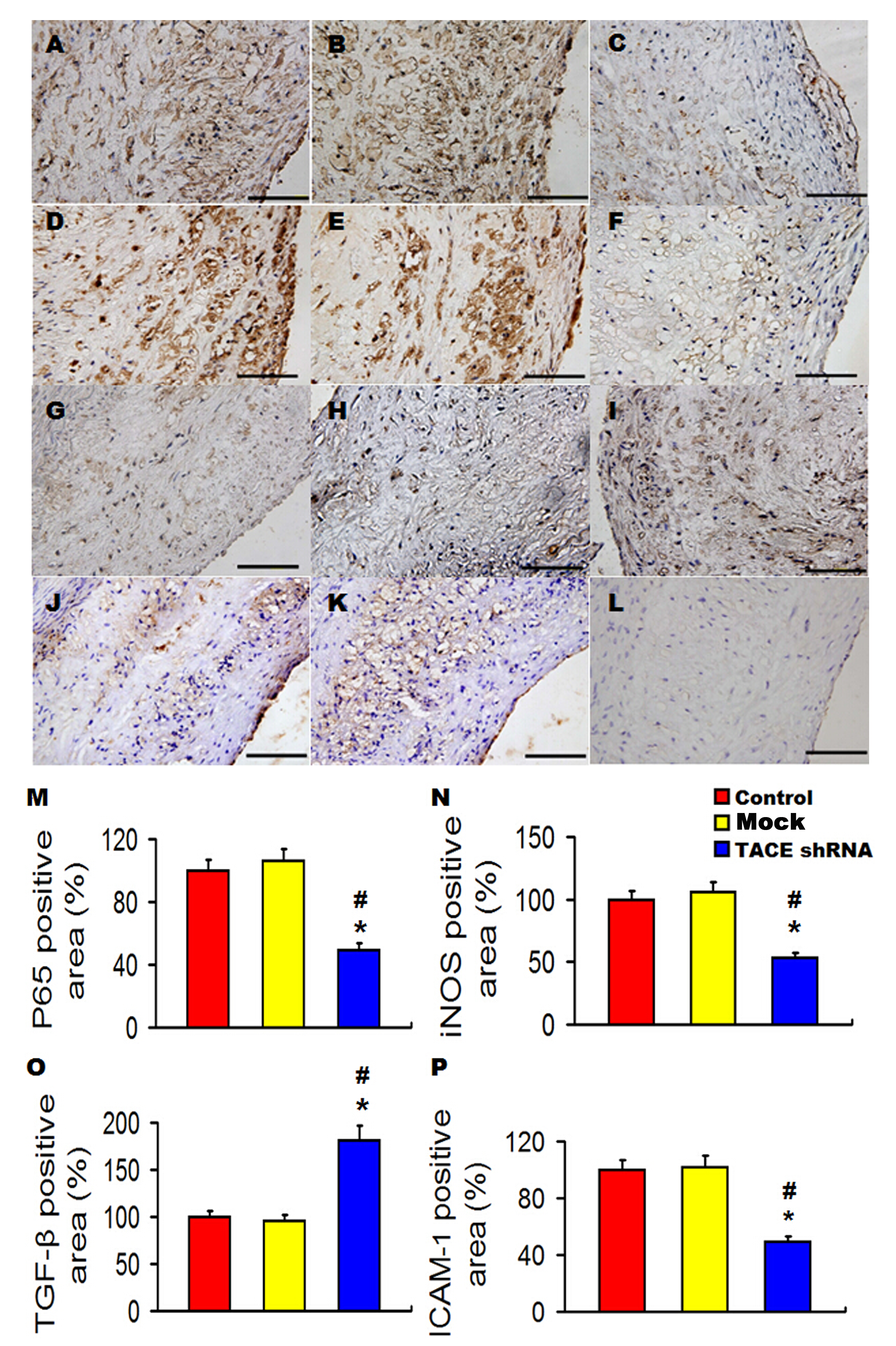


**Supplementary Figure 4. Effects of TACE gene silencing on inflammatory factor expression in the abdominal aortic plaques of three groups of rabbits.** (A)-(C) Representative immunostaining showing P65 expression in Control group (A), Mock group (B), and TACE shRNA group (C); (D)-(F) Representative immunostaining showing iNOS expression in Control group (D), Mock group (E), and TACE shRNA group (F); (G)-(I) Representative immunostaining showing TGF-β1 expression in Control group (G), Mock group (H), and TACE shRNA group (I); (J)-(L) Representative immunostaining showing ICAM-1 expression in Control group (J), Mock group (K), and TACE shRNA group (L); (M) Quantitative analysis of (A)-(C); (N) Quantitative analysis of (D)-(F); (O) Quantitative analysis of (G)-(I); (P) Quantitative analysis of (J)-(L). ﹡*P*<0.01 vs. Control group, ^#^*P* <0.01 vs. Mock group. Bar=100μm


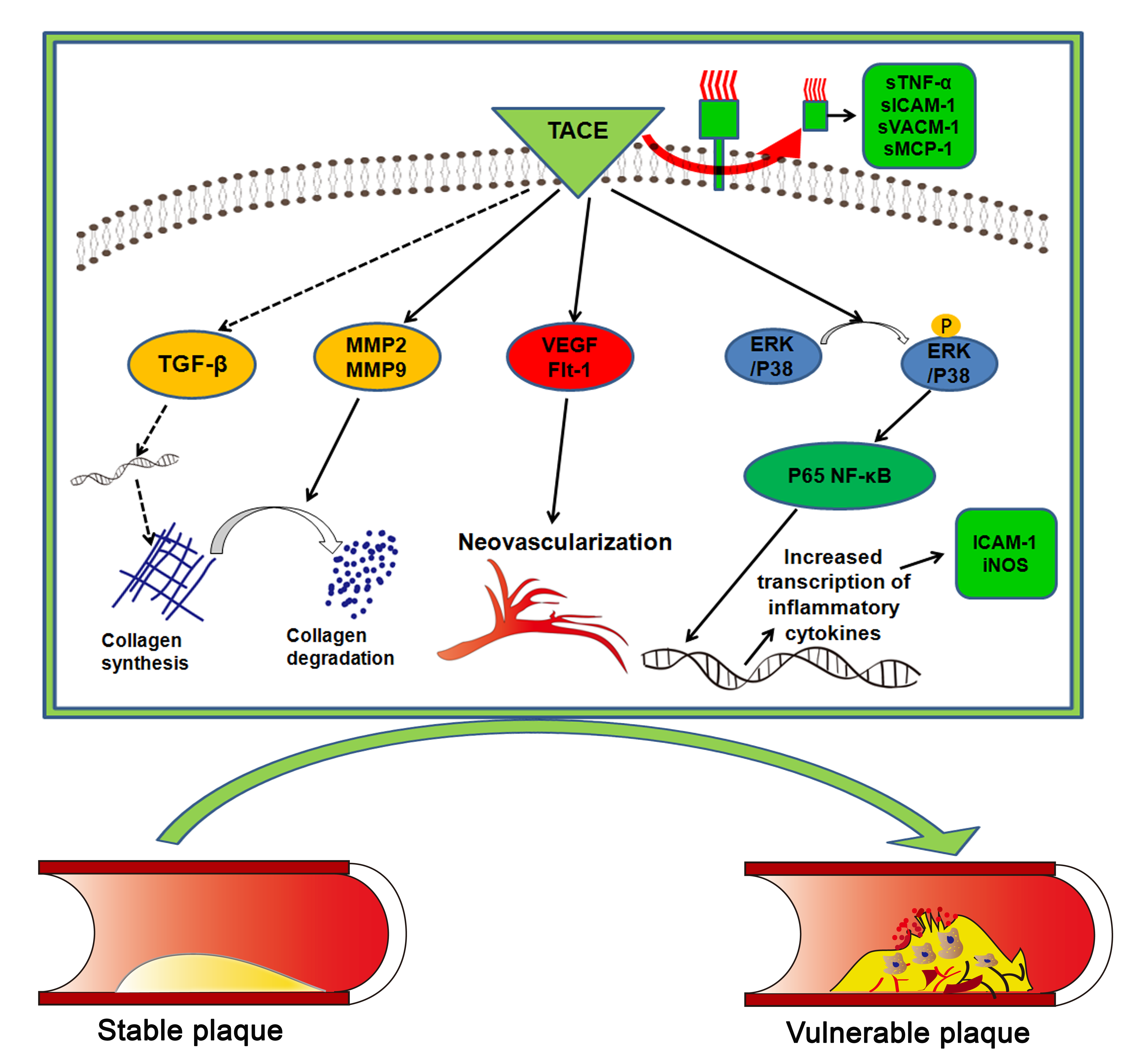


**Supplementary Figure 5. Potential mechanisms of the plaque destabilizing effects of TACE.** TACE may increase the serum levels of sTNF-α, sICAM-1, sVACM-1 and sMCP-1, leading to enhanced local inflammation. In addition, TACE may activate several intracellular signaling pathways including TGF-β, MMP2 and MMP9, ERK/P38/NF-κB, and VEGF, resulting in decreased collagen synthesis, increased collagen degradation, enhanced transcription of inflammatory cytokine genes, and spurred neovascularization in atherosclerotic lesions. These mechanisms may ultimately turn a stable plaque phenotype to a vulnerable plaque phenotype. The solid and dashed lines indicate stimulation and inhibition, respectively.

**
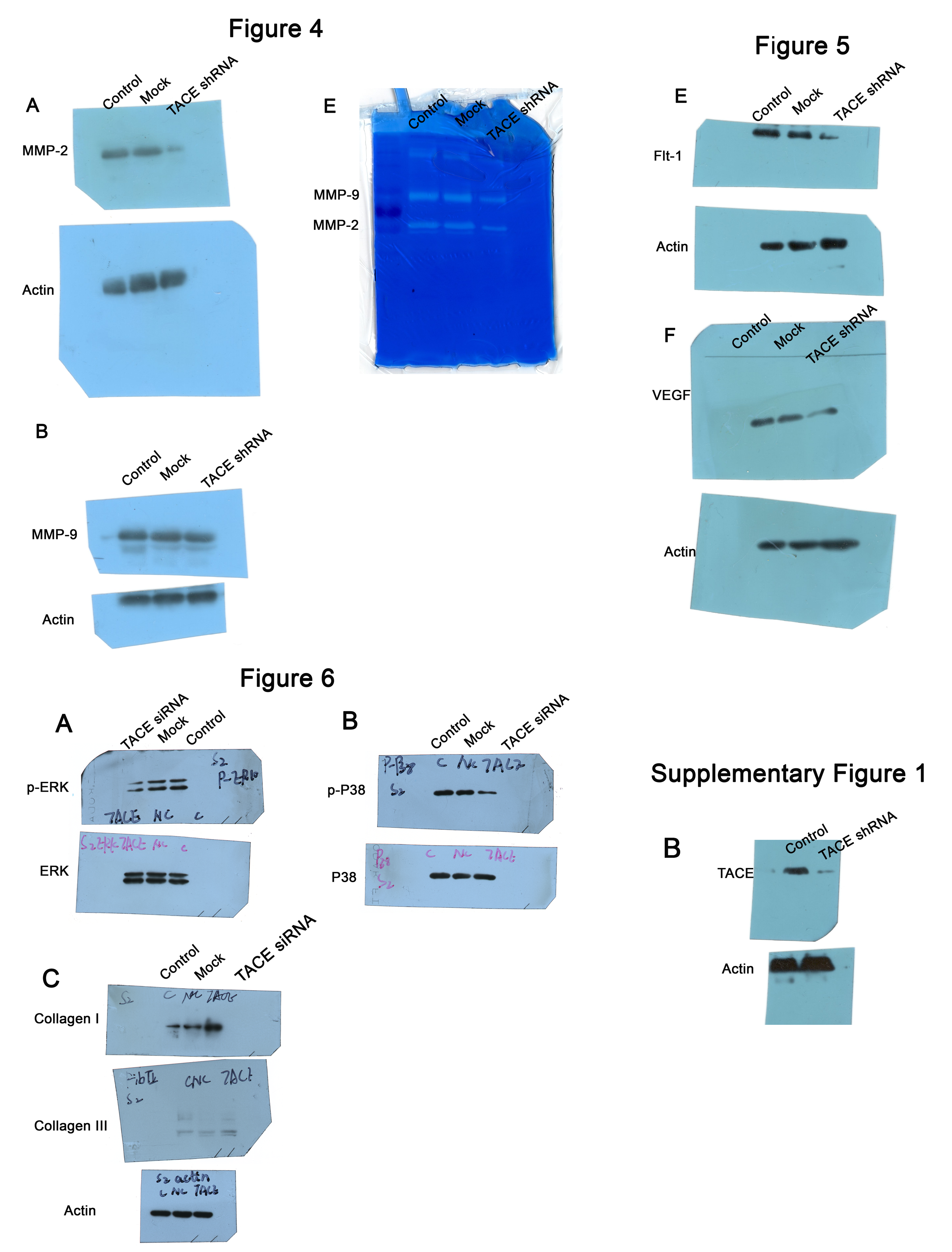
**

**Supplementary Figure 6. Full-length gels and blots.**
